# Optimization of Chitosan/Modified Chitosan–Silver(I) Composite Film and Application to Strawberries

**DOI:** 10.3390/foods15132312

**Published:** 2026-06-29

**Authors:** Jinhong Huang, Yiping Wang, Mengyuan Pang, Chengpeng Li, Pengzhi Hong, Zhang Hu

**Affiliations:** 1 Faculty of Chemistry and Environmental Science, Guangdong Ocean University, Zhanjiang 524088, China; hjh16607500604@163.com (J.H.); 18124470066@stu.gdou.edu.cn (Y.W.); 19914946070@163.com (M.P.); lcp0802@126.com (C.L.); 2 College of Food Science and Technology, Guangdong Ocean University, Zhanjiang 524088, China; hongpengzhigdou@163.com

**Keywords:** chitosan, composite film, antibacterial activity, fruit preservation

## Abstract

Fresh fruits are prone to spoilage due to microbial contamination and moisture loss, highlighting the need for effective packaging materials with strong barrier and antimicrobial functions. In this work, a bilayer composite film with antimicrobial and preservative properties was fabricated using the solution casting method, consisting of an inner chitosan (CS) layer and an outer complex layer of chitosan-2-pyridinecarboxaldehyde-Ag(I) (CS-PCA-Ag(I)). Preparation conditions were optimized via single-factor experiments combined with response surface methodology. The resulting composite film showed significantly enhanced mechanical properties, with tensile strength of 38.52 ± 2.07 MPa and elongation at break of 67.32 ± 1.47%, as well as a low water vapor permeability of 2.81 × 10^−7^ g·m^−1^·h^−1^·Pa^−1^. It also exhibited strong antibacterial activity against *Staphylococcus aureus* and *Escherichia coli*, with inhibition zone diameters of 19.8 ± 0.3 mm and 15.6 ± 0.2 mm, respectively. Strawberry preservation tests demonstrated that the CS/CS-PCA-Ag(I) film effectively suppressed microbial growth and reduced fruit weight loss (8.27 ± 0.42% after 5 days), thereby extending the shelf life of strawberries. Cytotoxicity evaluation and silver ion migration analysis further confirmed the film’s good biocompatibility and safety. Collectively, the CS/CS-PCA-Ag(I) composite film holds considerable promise for fresh food preservation applications.

## 1. Introduction

As a rich source of vitamins and various bioactive compounds, fresh fruits play an essential role in preserving human health and avoiding diseases [1]. However, postharvest fruits are highly susceptible to microbial contamination, oxidative reactions, and moisture loss, which lead to spoilage and quality deterioration, resulting in significant resource waste and economic losses [2]. Nearly one-third of fruits are lost every year as a result of microbial invasion and inadequate storage conditions, according to existing reports [3]. At present, strategies such as cold-chain transportation, steam heat treatment [4], irradiation sterilization [5], and the addition of chemical preservatives are commonly employed to extend fruit shelf life. Nevertheless, these approaches often suffer from high energy consumption or potential health risks associated with synthetic preservatives, making them inconsistent with the principles of green development and sustainability [6,7]. Hence, there has been growing interest in developing fruit preservation films that are safe, efficient, and environmentally friendly. The traditional petroleum-based preservation films primarily slow down quality deterioration through physical isolation; however, they are insufficient in effectively inhibiting microbial growth or preventing UV-induced quality degradation [8]. Moreover, their poor degradability may cause environmental pollution [9]. In contrast, food packaging films based on functional biopolymers exhibit excellent antibacterial, antioxidant, mechanical, and optical properties [10], and possess advantages such as renewability, biodegradability, and recyclability, receiving widespread attention in the field of food packaging.

Chitosan (CS) has broad prospects in the field of biofilms due to its excellent biodegradability, biocompatibility, low toxicity, broad-spectrum antibacterial activity, and good film-forming properties [11]. However, pure chitosan films suffer from limited mechanical strength, insufficient antibacterial activity, and poor water resistance, which restrict their practical applications [12]. Some studies demonstrated that molecular modification of CS and constructing CS-based composite systems can enhance their mechanical properties, thermal stability, antioxidant capacity, and UV-barrier performance, ultimately prolonging fruit shelf life [13,14].

To further enhance antibacterial performance, antimicrobial agents are often incorporated into chitosan-based materials. Silver ions (Ag^+^) demonstrate broad-spectrum antibacterial efficacy against both Gram-positive and Gram-negative bacteria [15]. Although silver nanoparticles (AgNPs) possess strong antibacterial properties, their practical application is hindered by some issues, such as aggregation, reduced active sites, and uncontrolled release of Ag^+^, which may induce potential cytotoxicity and compromise long-term antibacterial efficacy [16,17]. In comparison, silver complexes demonstrate greater structural stability and tunable release behavior, showing more promising application prospects in food packaging [18,19,20]. For example, CS-Ag composite systems have been reported to endow materials with excellent antibacterial activity and water absorption capacity, thereby delaying fruit spoilage [21].

Following our previous report on the synthesis and antibacterial properties of the CS-PCA-Ag(I) complex [22], this work endeavors to realize its utility as a food-packaging film. Yet, fabricating a film that simultaneously possesses mechanical durability, potent antibacterial activity, and good water resistance from this complex remains a technical challenge. Therefore, this study was designed to fabricate a novel composite bilayer film, strategically utilizing a chitosan (CS) layer as a structural support and a CS-PCA-Ag(I) layer as the functional antimicrobial interface. By employing response surface methodology to optimize the fabrication process, we aimed to develop a high-performance material that balances mechanical integrity with effective preservation functionality. The findings of this work provide a viable and sustainable strategy for the development of advanced chitosan-based packaging materials for postharvest fruit preservation.

## 2. Materials and Methods

### 2.1. Materials

Chitosan (degree of deacetylation ≥ 90%), 2-pyridinecarboxaldehyde (AR, 98%), silver nitrate (AR, 99.8%), and glacial acetic acid (AR, 99.5%) were supplied by Aladdin Biochemical Technology Co., Ltd. (Shanghai, China). Absolute ethanol and sodium hydroxide were purchased from Maclin Biochemical Technology Co., Ltd. (Shanghai, China). The L929 mouse fibroblast cell line (Serial: GNM28) was obtained from the Cell Bank of the Chinese Academy of Sciences (Shanghai, China). The bacterial strains *Escherichia coli* (*E. coil*, ATCC 8739) and *Staphylococcus aureus* (*S. aureus*, ATCC 6538) were obtained from the Guangdong Microbial Culture Collection Center (Guangzhou, China). Fresh strawberries were sourced from a local agricultural market in Zhanjiang, China. The 2-pyridinecarboxaldehyde-modified chitosan–silver complex (CS-PCA-Ag(I) complex) was prepared in our laboratory following the procedure described in our previous work [22]. All other chemicals were of analytical grade and used as received without further purification.

### 2.2. Preparation of CS/CS-PCA-Ag(I) Composite Films

The CS/CS-PCA-Ag(I) composite film was fabricated via the solution casting method. Briefly, chitosan was dissolved in a 1% (*v*/*v*) acetic acid solution, and an appropriate amount of glycerol was added as a plasticizer to form a uniform chitosan solution. Then, 10 mL of this solution was poured into a plastic film dish and dried at 40 °C for 6 h until a non-flowing, semi-dry matrix was formed. Afterwards, an equal volume of CS-PCA-Ag(I) solution was gently poured onto the surface of the chitosan layer and dried again at 40 °C until fully solidified, resulting in the CS/CS-PCA-Ag(I) bilayer composite film.

### 2.3. Single-Factor Experiments

To examine how formulation parameters affect the composite film properties, single-factor experiments were performed. The film performance was assessed based on elongation at break (EB) and water vapor permeability (WVP). Prior to the response surface optimization, preliminary experiments were conducted to determine the appropriate concentration ranges of CS, CS-PCA-Ag(I), and glycerol for the subsequent single-factor experiments and response surface analysis.

The mass fractions of chitosan (1.0%, 1.5%, 2.0%, 2.5%, and 3.0%), CS-PCA-Ag(I) (0.4%, 0.6%, 0.8%, 1.0%, and 1.2%), and glycerol (0.5%, 1.0%, 1.5%, 2.0%, and 2.5%) were investigated, respectively. During the single-factor experiments, only one factor was varied at a time, while the other two factors were maintained at constant levels. Specifically, when the CS concentration was varied, the concentrations of CS-PCA-Ag(I) and glycerol were fixed at 1.0% and 1.5%, respectively. When the concentration of CS-PCA-Ag(I) was varied, the concentrations of CS and glycerol were fixed at 2.0% and 1.5%, respectively. Similarly, when the glycerol concentration was varied, the concentrations of CS and CS-PCA-Ag(I) were fixed at 2.0% and 1.0%, respectively.

### 2.4. Box–Behnken Experimental Design

According to the single-factor experimental results, the mass fractions of chitosan (A), CS-PCA-Ag(I) (B), and glycerol (C) were chosen as independent variables, with elongation at break (EB, Y_1_) and water vapor permeability (WVP, Y_2_) as response variables. A three-factor, three-level Box–Behnken design was adopted to optimize the preparation conditions of the CS/CS-PCA-Ag(I) composite film. Design-Expert software (Version 13.0, Stat-Ease, Inc., Minneapolis, MN, USA) was used for experimental design and data analysis, and the interactions among factors, along with their significance levels, were analyzed using response surface methodology.

EB and WVP were selected as the response variables because the primary objective of the optimization was to achieve an appropriate balance between barrier performance and film flexibility. Tensile strength (TS) and antimicrobial activity were subsequently evaluated as important functional properties of the optimized films to verify the overall performance of the developed packaging material.

### 2.5. Fourier Transform Infrared Spectroscopy (FT-IR)

FT-IR spectra for the CS film, CS-PCA-Ag(I) film, and CS/CS-PCA-Ag(I) bilayer composite film were recorded on an FT-IR spectrometer (Nicolet iN10, Thermo Fisher Scientific, Waltham, MA, USA) equipped with an ATR accessory. The spectra were collected over a wavenumber range of 4000 to 600 cm^−1^ at a resolution of 4 cm^−1^ with 32 scans to evaluate the functional group modifications and chemical structures of the films.

### 2.6. Thermal Stability Analysis of the Films

The thermal degradation behavior of the CS film and the CS/CS-PCA-Ag(I) bilayer composite film was investigated using a simultaneous thermal analyzer (STA200, Hitachi, Japan) for thermogravimetric analysis and differential scanning calorimetry (TG-DSC). Film samples (approximately 8 mg) were placed in alumina crucibles and heated from 30 °C to 600 °C at a constant heating rate of 10 °C/min under a continuous nitrogen atmosphere with a flow rate of 20 mL/min.

### 2.7. Morphological Characterization

Scanning electron microscopy (SEM, MIRA LMS, TESCAN, Brno, Czech Republic) was used to examine the morphology of the CS/CS-PCA-Ag(I) composite films. For SEM observation, the samples were mounted on conductive tape and coated with a thin Au layer to increase conductivity. Atomic force microscopy (AFM, MultiMode 8, Bruker, Santa Barbara, CA, USA) analysis was conducted following the method reported by Gierszewska et al. [23] with minor modifications, and the surface morphology and roughness of the films were characterized in tapping mode. Nanoscope Analysis software (version 1.40) was used to scan and analyze randomly selected areas on the film surfaces. The roughness parameters (Ra, Rq, and Ssk, representing the arithmetic average roughness, root-mean-square roughness, and skewness of the film surface, respectively) were automatically calculated by the software based on the surface height deviations within the scanned areas according to ISO 4287 standards [24].

### 2.8. Mechanical Properties

The films were cut into rectangular samples (1 cm × 2 cm) and fixed between the grips of a universal testing machine (Rapid TA+, Bosin Industrial, Shanghai, China). The tensile strength and elongation at break were determined at a crosshead speed of 2 mm·min^−1^. Each sample was tested in triplicate. The thickness of each film was measured at five random positions using a digital micrometer, and the average value was used for the calculation of tensile strength. The average thicknesses of the CS, CS-PCA, and CS/CS-PCA-Ag(I) films used for mechanical property measurements were 0.087 ± 0.006 mm, 0.095 ± 0.004 mm, and 0.115 ± 0.009 mm, respectively.

### 2.9. Water Vapor Permeability (WVP)

The cup method, in accordance with ASTM E398-03 [25], was used to evaluate the water vapor barrier properties of the films. Specifically, anhydrous CaCl_2_ (0% RH) was placed into cups, which were then sealed with the film samples. The cup opening area and film thickness were recorded prior to testing. Each permeation cell was subsequently placed inside a desiccator containing saturated NaCl solution (75% RH). Weight gain was measured at 24 h intervals and plotted as a function of time (days). The water vapor transmission rate (WVTR, in g·m^−2^·day^−1^) was determined from the slope of the weight gain versus time plot. The WVP value was then computed using the equation below:

WVP = WVTR ∗ d/(T × Δp)

where WVTR represents water vapor transmission rate (g·m^−2^·day^−1^); T and d are the testing time (h) and the film thickness (m), respectively; and Δp is the partial vapor pressure difference between the atmosphere and pure water (Pa).

### 2.10. Antibacterial Activity

The agar diffusion method was employed to assess the antibacterial activity of the samples, using *Staphylococcus aureus* (Gram-positive) and *Escherichia coli* (Gram-negative) as the test strains. The culture medium was sterilized by autoclaving at 120 °C for 15 min. After the agar medium solidified, 100 μL of bacterial suspension (10^5^ CFU·mL^−1^) was added onto the agar surface and spread evenly with a sterile swab. Sterilized Oxford cups were placed on the agar plates with sterile tweezers, and 100 μL of film-forming solution with different concentrations was added to each cup. The plates were then incubated at (37 ± 2) °C for 24 h.

### 2.11. Application in Fruit Preservation

Fresh strawberries that were uniform in size and showed no mechanical damage were selected for the experiment. After being rinsed in deionized water for 10 min to eliminate surface contaminants, the strawberries were dried by wiping and then wrapped. In the experimental groups, CS film and CS/CS-PCA-Ag(I) composite film were used to wrap the strawberries, respectively, whereas commercial polyethylene film was applied to the control group. All wrapped samples were kept at 25 °C and 65% relative humidity over a 5-day period, during which the weight loss of strawberries was measured daily to evaluate the preservative efficacy of each film. The weight loss percentage was calculated according to the following equation:

Weight Loss (%) = (W_0_ − W_t_)/W_0_

where W_0_ is the initial weight of the strawberry sample (g), and W_t_ is the weight measured at a specific storage time (g).

### 2.12. Material Safety

#### 2.12.1. Cytotoxicity Assay

The cytotoxicity of the samples against mouse fibroblasts (L929 cells) was assessed via the CCK-8 assay. Film extracts were prepared by immersing the film samples in DMEM medium at an initial extraction concentration of 320 μg/mL and incubating at 37 °C for 24 h under sterile conditions. The extract solution was then filtered through a 0.22 μm sterile membrane and subsequently diluted with DMEM medium to the desired concentrations for cytotoxicity evaluation. Cells cryopreserved in liquid nitrogen were revived and subcultured twice before use. Logarithmic-phase cells were harvested, enumerated, and plated into 96-well plates, followed by incubation at 37 °C in a 5% CO_2_ atmosphere for 24 h. Afterwards, 20 μL of sample solutions at various concentrations were introduced and incubated for another 24 h. Next, 10 μL of CCK-8 solution was added to each well. After a 2 h incubation, the absorbance at 450 nm was recorded using a microplate reader (Synergy H1, Agilent Technologies, Inc., Santa Clara, CA, USA), and cell viability was determined.

#### 2.12.2. Determination of Silver Ion Release

The composite bilayer film was cut into 10 mm × 10 mm squares and floated on 10 mL of ultrapure water, with the chitosan (CS) layer facing downward in contact with the aqueous phase and the CS-PCA-Ag(I) layer oriented upward exposed to air. After standing at 25 °C for 24 h, the water sample was collected. The concentration of silver ions in the solution was determined using an atomic absorption spectrometer (AAS, iCE 3300, Thermo Fisher Scientific, Waltham, MA, USA). The measurement method was referred to Diridiri et al. [26] with slight modifications; briefly, a silver hollow cathode lamp was used as the radiation source, and the determination was performed at the primary resonance wavelength of 328.1 nm using an air-acetylene flame. This analysis evaluated the silver ion release behavior from the composite film.

### 2.13. Statistical Analysis

Unless otherwise specified, all experiments were performed in triplicate, and the results are expressed as mean ± standard deviations. The statistical analyses were conducted with SPSS 26.0 software. For comparisons between groups, independent sample t-tests were performed, and a probability value of *p* < 0.05 was considered statistically significant, and *p* < 0.01 was regarded as highly significant.

## 3. Results and Discussion

### 3.1. Single-Factor Experimental Results

#### 3.1.1. Effect of CS Content on the Properties of the Composite Film

**Figure 1:**
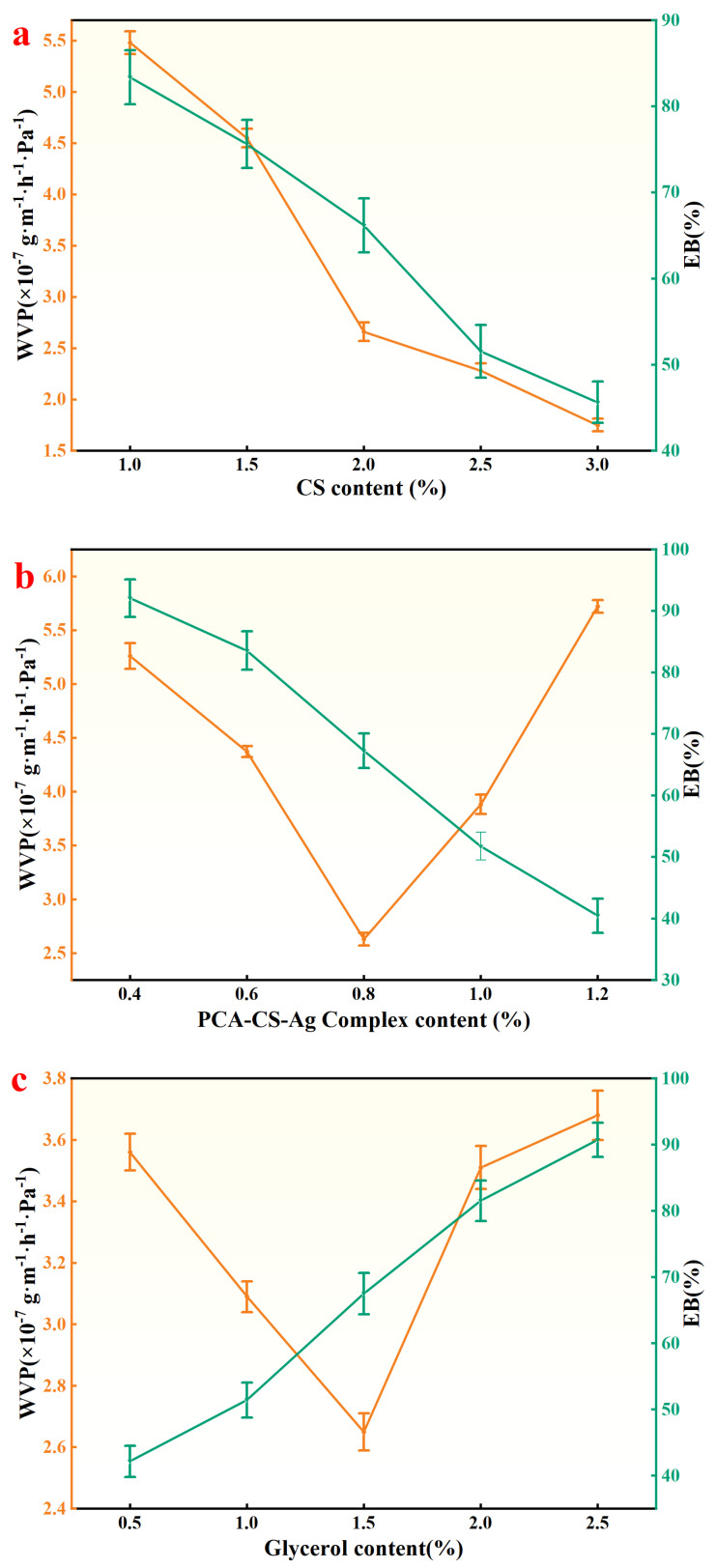
Influence of (**a**) CS content, (**b**) CS-PCA-Ag(I) content, and (**c**) glycerol content on the WVP and EB of CS/CS-PCA-Ag(I) composite films.

The effect of CS content on the properties of the composite film is presented in Figure 1a. When the CS content increased from 1.0% to 3.0% (*w*/*v*), the WVP value of the composite film decreased significantly from 5.48 ± 0.11 to 1.75 ± 0.06 (×10^−7^ g·m^−1^·h^−1^·Pa^−1^), which may be attributed to the enhanced hydrogen bonding interactions between the two layers with increasing CS content, resulting in a denser film structure that hindered water vapor diffusion [27]. Meanwhile, the elongation at break (EB) of the composite film decreased from 83.37 ± 3.15% to 45.62 ± 2.38% with increasing CS content, indicating that a higher CS content resulted in reduced film flexibility, which was consistent with previous reports by Jridi et al. [28] and Chen et al. [29].

#### 3.1.2. Effect of CS-PCA-Ag(I) Content on the Properties of the Composite Film

The effect of CS-PCA-Ag(I) content on the film properties was shown in Figure 1b. As its content increased from 0.4% to 0.8% (*w*/*v*), the WVP value of the composite film decreased from 5.26 ± 0.12 to 2.63 ± 0.06 (×10^−7^ g·m^−1^·h^−1^·Pa^−1^). However, when the content further increased to 1.2% (*w*/*v*), the WVP value sharply rose to 5.72 ± 0.06 (×10^−7^ g·m^−1^·h^−1^·Pa^−1^). Thus, an appropriate amount of CS-PCA-Ag(I) was conducive to improving the compactness of the film structure, thereby enhancing water vapor barrier performance, whereas excessive addition led to enhanced interactions between polymer chains, resulting in a heterogeneous film structure and increased water vapor permeability. Meanwhile, as CS-PCA-Ag(I) content increased from 0.4% to 1.2%, the EB of the composite film significantly decreased from 92.06 ± 3.07% to 40.45 ± 2.81%, indicating that a higher CS-PCA-Ag(I) content increased the brittleness and reduced the extensibility of the film.

#### 3.1.3. Effect of Glycerol Content on the Properties of the Composite Film

Glycerol was incorporated as an effective plasticizer to overcome the inherent brittleness of the CS-based matrix; it functions by inserting into the polymer network and disrupting the dense intermolecular hydrogen bonds between chains, thereby increasing the free volume and molecular mobility [30]. Notably, a lack or excess of glycerol would compromise the film’s integrity, which necessitates an optimized concentration [31]. The effect of glycerol content on the properties of the composite film was presented in Figure 1c. As the glycerol content increased from 0.5% to 1.5% (*v*/*v*), the WVP of the composite film decreased from 3.56 ± 0.06 to 2.65 ± 0.06 (×10^−7^ g·m^−1^·h^−1^·Pa^−1^); whereas further increase to 2.5%, the WVP increased to 3.68 ± 0.08 (×10^−7^ g·m^−1^·h^−1^·Pa^−1^). As for the effect on the EB of the composite film, with increasing glycerol content, the EB significantly increased from 42.15 ± 2.33% to 90.72 ± 2.57%, which was attributed to the fact that glycerol, as a plasticizer, can weaken the interactions between polymer chains and enhance the mobility of molecular chains, thereby significantly improving the flexibility of films [32].

### 3.2. Preparation Optimization of the Composite Film by Response Surface Methodology

#### 3.2.1. Box–Behnken Design and ANOVA Analysis

**Figure 2:**
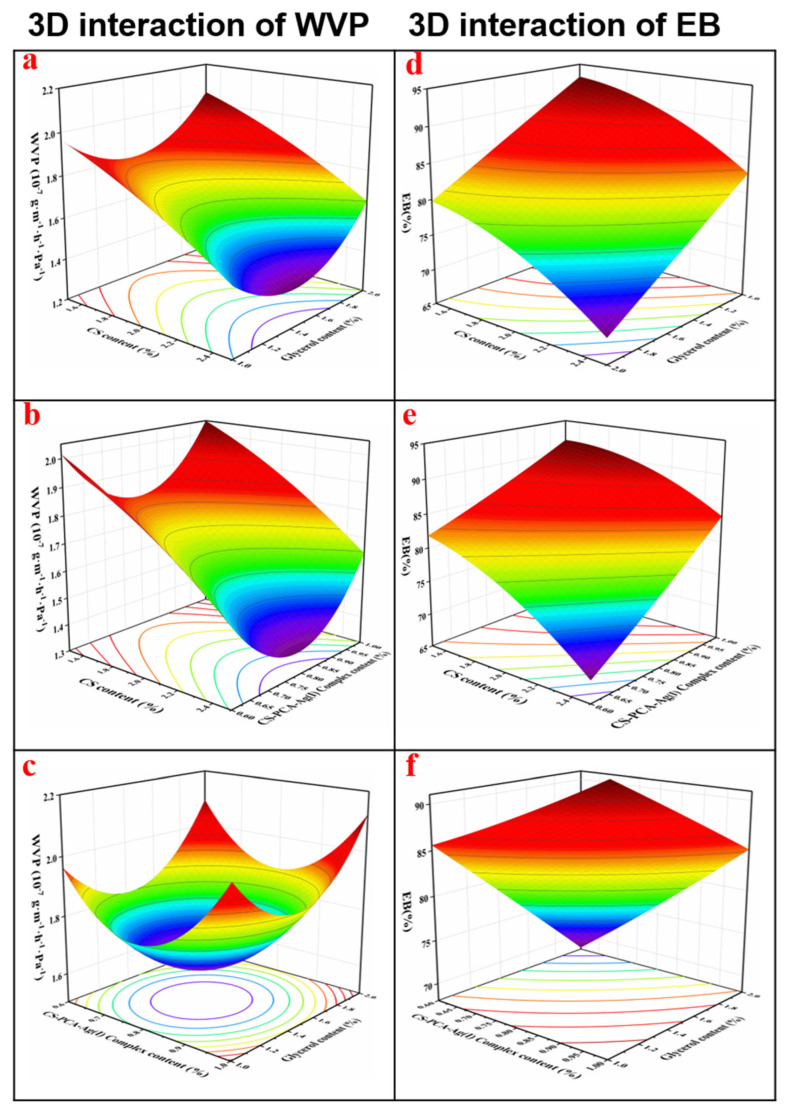
3D interaction plots of WVP (**a**–**c**) and EB (**d**–**f**): (**a**,**d**) CS and glycerol content; (**b**,**e**) CS and CS-PCA-Ag(I) content; (**c**,**f**) CS-PCA-Ag(I) and glycerol content. The color transition from red to blue/purple represents high to low values, respectively.

According to the single-factor experimental results, a Box–Behnken response surface design was adopted to optimize the composite film preparation conditions, with CS content (A), CS-PCA-Ag(I) content (B), and glycerol content (C) as independent variables, and WVP and EB as response variables. Seventeen experimental runs were generated, and the results are shown in Table 1. The experimental data were analyzed using Design-Expert 13 software, and the relationships between variables and responses were visualized through three-dimensional response surface plots (Figure 2).

The ANOVA results for WVP are presented in Table 2. The model was highly significant (<0.0001), indicating that the regression model adequately described the experimental data. The linear terms A, B, and C had significant effects on WVP (*p* < 0.0001), and the interaction between B and C was also significant (*p* = 0.0395). The lack of fit was not significant (*p* = 0.1392), suggesting that the model provided a satisfactory fit to the experimental results. In addition, the coefficient of determination (R^2^ = 0.9993) confirmed the high reliability of the model.

The ANOVA outcomes for EB are summarized in Table 3. The model was highly significant (p<0.0001), with each of the linear factors A, B, and C exerting a significant influence on EB (p<0.0001). Interactive effects were also significant for AB (p=0.0457) and BC (p=0.0173), indicating that the interplay between these variables substantially affected film flexibility. The lack-of-fit term was insignificant (p=0.2866), and the high determination coefficient ($R^{2}=0.9950$) confirmed the reliability of the regression model.

#### 3.2.2. Response Surface Analysis and Optimization

The experimental results were fitted to quadratic polynomial equations describing the relationships between the independent variables and the responses. The regression models for WVP and EB were expressed as follows:

WVP = 22.70 + 0.13A − 32.32B − 8.29C + 0.23AB + 0.05AC − 0.43BC − 0.48A^2^ + 20.63B^2^ + 2.98C^2^

EB = 123.06 + 14.40A − 48.31B − 47.63C + 16.35AB + 1.84AC + 20.90BC − 11.88A^2^ + 22.64B^2^ + 1.58C^2^

To obtain a balanced performance between barrier and mechanical properties, the desirability function in Design-Expert 13 was used to optimize the preparation conditions with equal weights assigned to WVP and EB (50% each). The optimal formulation was predicted as CS content 2.39%, CS-PCA-Ag(I) content 0.88%, and glycerol content 1.17%. Under these conditions, the predicted WVP and EB values were 2.75 (×10^−7^ g·m^−1^·h^−1^·Pa^−1^) and 69.08%, respectively.

Three parallel experiments were performed under the optimized conditions to test the model’s reliability. The resulting WVP and EB values were determined to be 2.81 (×10^−7^ g·m^−1^·h^−1^·Pa^−1^) and 67.32%, which deviated by 2.13% and 2.54% from the predicted values, respectively. Thus, the response surface model proved to be accurate and reliable in predicting the preparation conditions of the composite films.

The optimized formulation (CS content 2.39%, CS-PCA-Ag(I) content 0.88%, and glycerol content 1.17%) was selected for the preparation of the CS/CS-PCA-Ag(I) composite film. The prepared bilayer composite film was subsequently used for all subsequent characterizations and performance evaluations.

### 3.3. FT-IR Analysis

**Figure 3:**
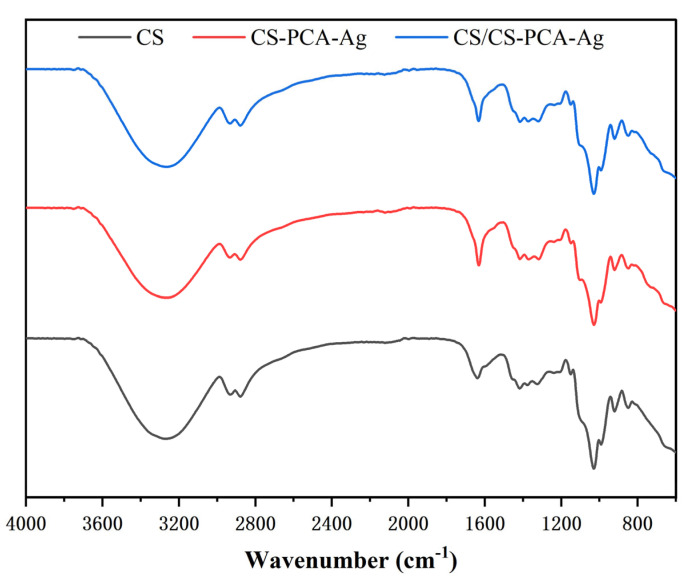
FT-IR of CS, CS-PCA-Ag, and CS/CS-PCA-Ag films.

The FT-IR spectra of CS, CS-PCA-Ag(I), and CS/CS-PCA-Ag(I) films are shown in Figure 3. All films exhibited a broad absorption band around 3260–3270 cm^−1^, which was attributed to the overlapping stretching vibrations of O–H and N–H groups. The broadening of this band was associated with hydrogen-bonding interactions involving glycerol [33]. Compared with CS, the spectra of CS-PCA-Ag(I) and CS/CS-PCA-Ag(I) films exhibited a shifted absorption band near 1630 cm^−1^, indicating the formation of imine (C=N) groups through the Schiff-base reaction between chitosan and pyridine-2-carboxaldehyde [34]. In addition, the slight shift in the characteristic band suggested coordination interactions between Ag(I) and the nitrogen-containing functional groups of the CS-PCA matrix [22]. Since the ATR-FTIR spectrum of the bilayer film was collected from the CS-PCA-Ag(I) surface, its spectral features were similar to those of the CS-PCA-Ag(I) film. Minor changes in peak position and intensity indicated possible intermolecular interactions between the CS and CS-PCA-Ag(I) layers.

### 3.4. TG-DSC Analysis

**Figure 4:**
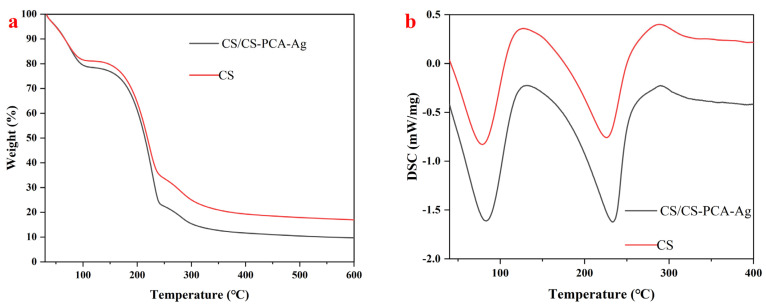
Thermal analysis of pure CS and CS/CS-PCA-Ag(I) bilayer composite films: (**a**) TG curves and (**b**) DSC curves.

The thermal stability of the pure CS film and the CS/CS-PCA-Ag(I) bilayer composite film was evaluated via simultaneous TG-DSC analysis (Figure 4). The initial weight loss observed between 30 °C and 100 °C in TG curves (Figure 4a) corresponds to the evaporation of free and bound water within the hydrophilic polymer matrices [35]. This dehydration process is perfectly reflected by the broad endothermic peaks below 100 °C in the DSC profiles (Figure 4b). The second major degradation stage (150–350 °C) is dominated by the depolymerization and pyrolytic decomposition of the chitosan backbone. In our previous study, the chemical modification of the single-layer CS-PCA-Ag matrix disrupted the rigid intermolecular hydrogen-bonding network, compromising its thermal tolerance and dropping its onset degradation temperature to 193 °C [22]. In contrast, the newly developed CS/CS-PCA-Ag(I) bilayer film exhibits significantly enhanced thermal stability, with its onset degradation temperature shifting to approximately 240 °C. This thermal enhancement is directly confirmed by the primary decomposition peaks in both TG and DSC curves moving toward higher temperatures. This remarkable improvement is attributed to the unique bilayer architecture. The pristine CS outer layer, possessing high crystallinity and a dense macromolecular network, effectively acts as a robust thermal protective barrier that restricts polymer segment motion and retards heat transfer into the inner active layer. Consequently, this elevated thermal threshold confirms that the CS/CS-PCA-Ag(I) bilayer film possesses excellent thermal endurance, safely meeting the safety and processing requirements for food-packaging applications.

### 3.5. Morphological Analysis

**Figure 5:**
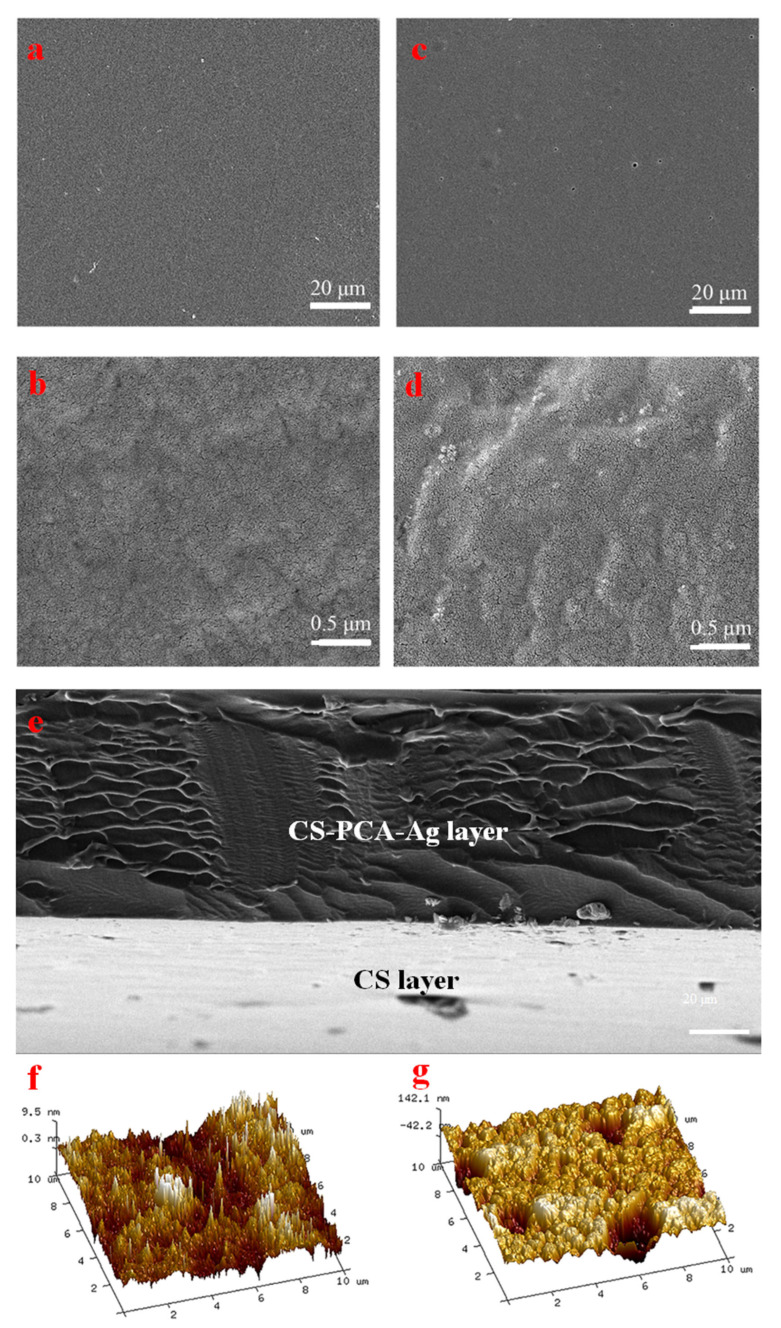
SEM images and AFM analysis of CS/CS-PCA-Ag(I) composite films. (**a**,**b**) SEM images of the CS layer surface; (**c**,**d**) SEM images of the CS-PCA-Ag(I) layer surface; (**e**) cross-sectional structure of the CS/CS-PCA-Ag(I) composite films; (**f**,**g**) AFM topography and surface roughness analysis of CS and CS-PCA-Ag(I) layers.

SEM was used to characterize the surface and cross-sectional morphologies of the optimized CS/CS-PCA-Ag(I) composite film prepared under the response surface optimized conditions, as presented in Figure 5. Both sides of the films exhibited relatively smooth surfaces without obvious wrinkles or cracks. The cross-sectional images revealed a distinct structure. The CS layer displayed a relatively smooth morphology (Figure 5a,b), whereas several fine silver-containing particles were observed on the CS-PCA-Ag(I) layer (Figure 5c,d). These particles may be associated with the incorporation and distribution of silver species within the CS-PCA matrix. Although SEM analysis confirmed the presence of silver species in the composite film, the morphology and crystallographic nature of the observed silver-containing particles cannot be conclusively determined without complementary analyses such as X-ray photoelectron spectroscopy (XPS) or X-ray diffraction (XRD). Additionally, the cross-sectional images exhibited a distinct interface between the two layers, and the CS layer adhered tightly to the CS-PCA-Ag(I) layer without noticeable delamination, suggesting favorable interfacial compatibility between the two layers (Figure 5e). This observation is further supported by the FTIR, TG-DSC, and mechanical property results, which suggest the existence of intermolecular interactions between the CS and CS-PCA-Ag(I) layers.

Further comparison of the microstructural differences between the two surfaces was performed using atomic force microscopy (AFM, Dimension ICON, Bruker, Santa Barbara, CA, USA) (Figure 5f,g). The two sides of the composite film exhibited distinctly different roughness levels. The CS side was relatively smooth, with an average roughness (Ra) of 1.91 nm and a root mean square roughness (Rq) of 2.50 nm. By contrast, the CS-PCA-Ag(I) side displayed a more uneven surface, with considerably higher roughness values (Ra = 32.7 nm, Rq = 46.1 nm). Additionally, the skewness value (Ssk) of the CS layer surface was close to zero, indicating that the number of micropit arrays and the number of micropillar arrays on the film surface were nearly equivalent, which contributed to the formation of a relatively smooth surface, whereas the CS-PCA-Ag(I) layer exhibited a negatively skewed surface (Ssk = −1.45) dominated by micropit arrays. Wang et al. [36] demonstrated that negatively skewed surfaces with micropit arrays are more likely to promote droplet splashing. This phenomenon is mainly attributed to the formation of a Cassie–Baxter state on negatively skewed surfaces.

### 3.6. Mechanical Property Analysis

**Figure 6:**
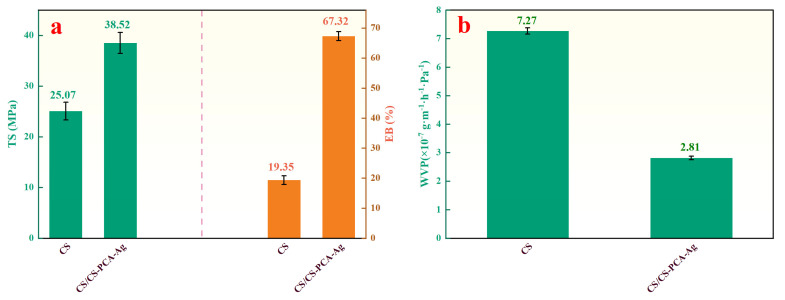
Evaluation of mechanical properties and water vapor permeability for CS-PCA and CS/CS-PCA-Ag(I) composite films. Panel (**a**) presents tensile strength (TS) and elongation at break (EB); panel (**b**) shows water vapor permeability (WVP).

Mechanical properties directly govern the resistance to breakage, seal integrity, and barrier performance of food packaging films during food processing, sealing, transport, and storage. Therefore, mechanical performance is considered one of the essential parameters for evaluating food packaging film materials. The mechanical properties of the CS film, CS-PCA film, and CS/CS-PCA-Ag(I) composite film are presented in Figure 6a. For the pure CS film, the tensile strength (TS) was 25.07 ± 1.73 MPa, and the elongation at break (EB) was 19.35 ± 1.46%. Compared with the CS film, the TS of the CS-PCA film slightly decreased, whereas the EB significantly increased (*p* < 0.01), which may be attributed to the enhanced flexibility of polymer chains after modification, resulting in improved film extensibility. A similar mechanical behavior has been reported for succinylated chitosan films [37]. In contrast, the CS/CS-PCA-Ag(I) composite film exhibited significantly improved mechanical properties, with TS and EB values of 38.52 ± 2.07 MPa and 67.32 ± 1.47%, respectively, both of which were significantly higher than those of the pure CS film (*p* < 0.01). The enhancement in mechanical performance may be attributed to the chelation interactions between silver ions and modified chitosan, as well as the partial penetration of CS-PCA-Ag(I) into the CS matrix during film formation, which resulted in a more compact structure and strengthened intermolecular interactions. This structural reinforcement effect was similar to the modification of composite films induced by silver nanoparticles [38,39].

### 3.7. WVP Analysis

WVP is a key parameter for evaluating the moisture barrier properties of film materials. The WVP results of the prepared films are presented in Figure 6b. The WVP value of the pure CS film was 7.27 × 10^−7^ g·m^−1^·h^−1^·Pa^−1^, which was comparable to the values reported by Zhang et al. [40] (0.55 g·mm·h^−1^·kPa^−1^·m^−2^). In contrast, the CS/CS-PCA-Ag(I) composite film exhibited a significantly lower WVP value (2.81 × 10^−7^ g·m^−1^·h^−1^·Pa^−1^), indicating improved water vapor barrier performance, which is particularly important for fruit preservation, as films with lower WVP can effectively reduce excessive moisture loss and prevent the entry of external microorganisms. The lower WVP of the CS/CS-PCA-Ag(I) composite film is ascribed to the chelation between Ag^+^ ions and PCA-modified chitosan. Additionally, partial penetration of CS-PCA-Ag(I) into the CS layer during film formation led to the formation of a more compact structure. This dense structure not only restricted the interaction between CS and water molecules but also exerted a crosslinking-like effect, thereby further reducing water vapor permeability [20].

### 3.8. Antibacterial Properties

**Figure 7:**
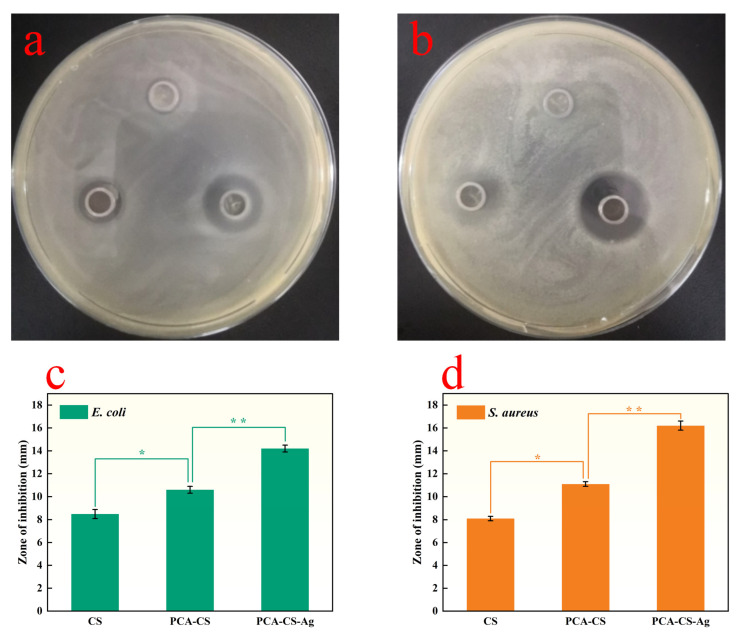
Antibacterial properties of CS, CS-PCA, and CS-PCA-Ag(I) films against *S. aureus* and *E. coli*. (**a**,**b**) Inhibition zone photos. (**c**,**d**) Corresponding inhibition zone diameters. Data are expressed as mean ± standard deviation (* *p* < 0.05, and ** *p* < 0.01).

The antibacterial efficacy of the CS, CS-PCA, and CS-PCA-Ag(I) films against *S. aureus* and *E. coli* is illustrated in Figure 7. Only limited inhibitory activity was observed for the CS film, producing inhibition zones of 10.3 ± 0.2 mm (against *S. aureus*) and 9.1 ± 0.2 mm (against *E. coli*). Such weak antibacterial performance of the CS film arises from a combination of intrinsic parameters (e.g., origin, concentration, molecular weight, deacetylation degree, and polymerization degree) and extrinsic conditions (e.g., pH of the environment, microbial strain, and susceptibility), as noted in previous work [41]. After a grafting modification, the CS-PCA film yielded distinctly larger inhibition zones of 13.5 ± 0.3 mm for *S. aureus* and 11.3 ± 0.2 mm for *E. coli*, both surpassing those of unmodified CS. This improvement was likely attributable to the presence of imine moieties (–C=N–) within the modified structure, which promoted its permeability, hydrophilicity, solubility, positive charge density, and metal coordination—collectively boosting the antimicrobial effect—in agreement with earlier reports [42,43]. Upon chelation with Ag^+^, the CS-PCA-Ag(I) gave inhibition zone diameters of 19.8 ± 0.3 mm (*S. aureus*) and 15.6 ± 0.2 mm (*E. coli*), and a statistically significant increase (*p* < 0.01) relative to CS-PCA film was observed.

**Figure 8:**
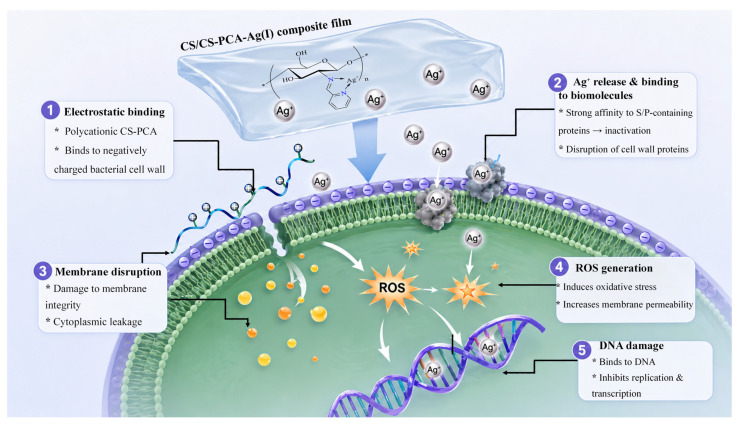
Schematic illustration of the antimicrobial mechanism of the CS/CS-PCA-Ag(I) bilayer composite film.

The proposed antibacterial mechanism of the CS-PCA-Ag(I) film was primarily attributed to the synergistic effect between CS-PCA and silver species incorporated within the film matrix (Figure 8). Chitosan’s polycationic nature enabled electrostatic binding to negatively charged bacterial cell walls, disrupting membrane integrity and causing cytoplasmic leakage [44]. The introduction of imine groups by grafting PCA onto CS further strengthened the hydrophilicity and positive charge density of CS, thereby facilitating stronger interactions with cellular components [45,46]. In addition, silver species coordinated within the CS-PCA matrix may contribute to antibacterial activity through direct interactions with bacterial cells. Although the overall migration of Ag^+^ from the film was extremely low, trace amounts of silver ions potentially present at the film–bacteria interface may still participate in membrane disruption, enzyme inactivation, oxidative stress induction, and interference with DNA replication and transcription [47,48]. Owing to the improved hydrophilicity and positive charge density of CS-PCA, microorganisms can readily adhere to the polymer matrix, facilitating close contact between bacterial cells and the active components within the film. Therefore, the enhanced antibacterial activity of the CS-PCA-Ag(I) film is likely attributable to the synergistic action of CS-PCA and silver species incorporated within the film matrix [49].

### 3.9. Fruit Preservation Application

**Figure 9:**
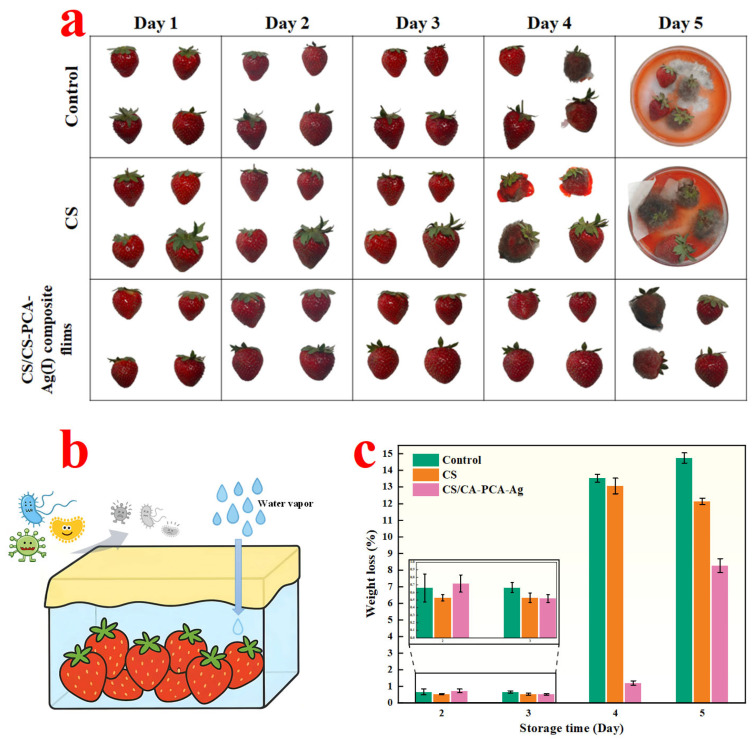
Preservation performance of different films on strawberries. (**a**) Photographs of strawberries stored under different packaging films over 5 days; (**b**) schematic illustration of the preservation mechanism of the CS/CS-PCA-Ag(I) composite film; (**c**) weight loss rates of strawberries after 5 days of storage.

Strawberries are highly perishable due to their soft texture, high respiration and transpiration rates, and high susceptibility to fungal spoilage, resulting in a relatively short shelf life [50]. The preservation performance of strawberries under different packaging films is illustrated in Figure 9a. Strawberries stored in trays covered with pure CS film and conventional PE film exhibited visible spoilage within 3 days, accompanied by mycelium formation on the surface, and severe dehydration became apparent on the fourth day. In contrast, strawberries packaged with the CS/CS-PCA-Ag(I) composite film exhibited only slight mycelial growth on the fifth day, indicating a significantly improved preservation effect. Moreover, the weight loss rate of strawberries packaged with the CS/CS-PCA-Ag(I) composite film after 5 days of storage was 8.27 ± 0.42%, which was significantly lower than those of the pure CS film (12.13 ± 0.79%) and PE film (14.74 ± 0.30%) (Figure 9c). These results indicate that the CS/CS-PCA-Ag(I) composite film effectively reduces moisture loss and preserves fruit quality during storage. A similar preservation effect has been reported for chitosan-based composite films developed by Qian et al. [51]. As depicted in Figure 9b, the preservation mechanism of the CS/CS-PCA-Ag(I) composite film may be attributed to two primary factors. On one hand, the relatively low water vapor permeability of the composite film contributes to reducing moisture loss from the fruit. On the other hand, the amino groups and Ag^+^ within the composite film can disrupt microbial cell structures and suppress microbial proliferation, thereby shielding the fruit from external microbial contamination and consequently prolonging its shelf life. Therefore, the CS/CS-PCA-Ag(I) composite film exhibits considerable potential for preventing fruit spoilage and extending the storage life of fresh fruits.

### 3.10. Safety Evaluation

#### 3.10.1. Cytotoxicity

**Figure 10:**
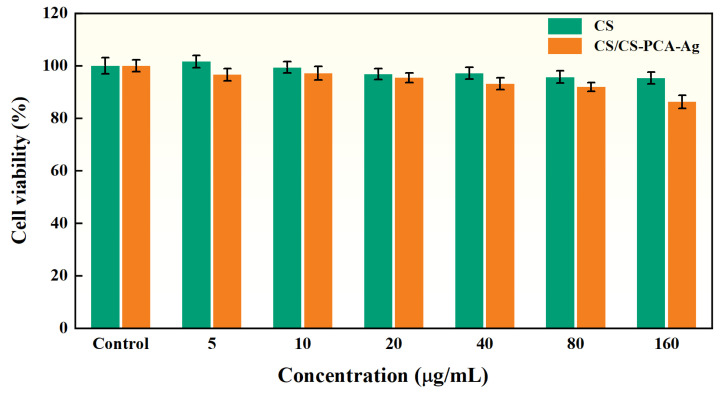
In vitro cytocompatibility of pure CS and CS/CS-PCA-Ag(I) composite films toward L929 cells evaluated by CCK-8 assay.

Good biocompatibility is an important requirement for food packaging materials. The cytocompatibility of the prepared films was evaluated in vitro using L929 cells via the CCK-8 assay. As shown in the figure, owing to the inherent biocompatibility of chitosan, the pure CS film exhibited negligible cytotoxicity compared with the control, which is consistent with previous reports [52]. For the CS/CS-PCA-Ag(I) composite film, no significant inhibition of L929 cell proliferation was observed within the concentration range of 5.0–80.0 μg/mL. Even at a higher concentration of 160.0 μg/mL, the cell viability remained at 86.25%, which still falls within the acceptable safety range for biomaterials [53]. These results indicated that the CS/CS-PCA-Ag(I) composite film exhibited no cytotoxicity toward L929 cells in vitro and demonstrated good cytocompatibility (Figure 10). Furthermore, considering the structure of the CS/CS-PCA-Ag(I) film, the CS-PCA-Ag(I) layer is situated on the outer layer and does not have direct contact with food, further enhancing its safety in food packaging applications.

#### 3.10.2. Evaluation of Material Safety

After the chitosan layer of the CS/CS-PCA-Ag(I) film had been kept in contact with the water surface for 24 h, the concentration of silver ions released into the solution was determined by atomic absorption spectroscopy. The concentration of Ag^+^ was below the detection limit of the instrument (<0.01 mg/L), indicating that the CS-PCA-Ag(I) complex was relatively stable without directly contacting water. This property not only maintains the antimicrobial activity of Ag^+^ but also improves the safety of the material. However, according to the Commission Regulation (EU) No 10/2011 issued by the European Commission in 2011 [54], additional safety evaluations are still required before the material can be applied directly in food contact applications.

## 4. Conclusions

In this study, the CS/CS-PCA-Ag(I) composite film was successfully fabricated, and its structural characteristics and functional properties were investigated. The results demonstrated that the composite film possessed a well-defined bilayer structure with good interfacial compatibility. Relative to the pure CS film, the bilayer composite film exhibited substantially enhanced mechanical properties and reduced water vapor permeability, reflecting improved mechanical stability and barrier performance. Moreover, the CS/CS-PCA-Ag(I) composite film showed strong antibacterial activity against *E. coli* and *S. aureus*, primarily attributable to the synergistic antibacterial effects between modified chitosan and silver species incorporated within the film matrix. The strawberry preservation experiment further confirmed that the bilayer composite film effectively reduced moisture loss and delayed fruit spoilage, thereby extending the shelf life of strawberries. However, additional quality indicators, including firmness, soluble solid content, titratable acidity, vitamin C content, color parameters, and microbial counts, as well as different storage temperatures and storage durations, should be investigated in future studies to provide a more comprehensive evaluation of its practical application potential. The safety evaluation revealed that the composite film had good cytocompatibility toward L929 cells and that the release of silver ions was controllable. Collectively, the CS/CS-PCA-Ag(I) composite film holds great promise for applications in food packaging and postharvest fruit preservation.

## Figures and Tables

**Table 1:** The experimental design and results of response surface methodology.

Runs	A CS Content (%)	B CS-PCA-Ag(I) Complex Content (%)	C Glycerol Content (%)	Response 1 WVTR (×10^−7^ g·m^−1^·h^−1^·Pa^−1^)	Response 2 EB (%)
1	1.5	0.6	1.5	4.08	65.78
2	2.5	0.6	1.5	2.48	47.12
3	1.5	1.0	1.5	4.19	83.61
4	2.5	1.0	1.5	2.68	71.49
5	1.5	0.8	1.0	3.85	87.84
6	2.5	0.8	1.0	2.31	67.77
7	1.5	0.8	2.0	4.22	64.29
8	2.5	0.8	2.0	2.73	46.06
9	2.0	0.6	1.0	3.84	73.39
10	2.0	1.0	1.0	4.17	89.68
11	2.0	0.6	2.0	4.36	46.87
12	2.0	1.0	2.0	4.52	71.52
13	2.0	0.8	1.5	2.62	68.43
14	2.0	0.8	1.5	2.67	69.55
15	2.0	0.8	1.5	2.64	70.59
16	2.0	0.8	1.5	2.65	67.51
17	2.0	0.8	1.5	2.68	69.24

**Table 2:** ANOVA analysis for WVP.

Source	Sum of Squares	df	Mean Square	F-Value	*p*-Value
Model	10.65	9	1.18	1044.08	<0.0001
A	4.71	1	4.71	4159.79	<0.0001
B	0.0800	1	0.0800	70.62	<0.0001
C	0.3445	1	0.3445	304.05	<0.0001
AB	0.0020	1	0.0020	1.79	0.2230
AC	0.0006	1	0.0006	0.5517	0.4818
BC	0.0072	1	0.0072	6.38	0.0395
A^2^	0.0604	1	0.0604	53.30	0.0002
B^2^	2.87	1	2.87	2531.23	<0.0001
C^2^	2.34	1	2.34	2064.26	<0.0001
Residual	0.0079	7	0.0011		
Lack of Fit	0.0056	3	0.0019	3.30	0.1392
Pure Error	0.0023	4	0.0006		
R^2^				0.9993	

**Table 3:** ANOVA analysis for EB.

Source	Sum of Squares	df	Mean Square	F-Value	*p*-Value
Model	2540.53	9	282.28	155.32	<0.0001
A	596.51	1	596.51	328.21	<0.0001
B	864.03	1	864.03	475.41	<0.0001
C	1011.15	1	1011.15	556.36	<0.0001
AB	10.69	1	10.69	5.88	0.0457
AC	0.8464	1	0.8464	0.4657	0.5169
BC	17.47	1	17.47	9.61	0.0173
A^2^	37.13	1	37.13	20.43	0.0027
B^2^	3.45	1	3.45	1.90	0.2106
C^2^	0.6586	1	0.6586	0.3624	0.5662
Residual	12.72	7	1.82		
Lack of Fit	7.31	3	2.44	1.80	0.2866
Pure Error	5.41	4	1.35		
R^2^				0.9950	

## Data Availability

The original contributions presented in this study are included in the article. Further inquiries can be directed to the corresponding author.

## References

[1] Di Giosia P., Stamerra C.A., Giorgini P., Jamialahamdi T., Butler A.E., Sahebkar A. (2022). The role of nutrition in inflammaging. Ageing Res. Rev..

[2] Zhang J., Cai C., Hu Y., Tan Z. (2025). An active packaging film prepared from aqueous two-phase Pickering emulsion and chitosan for fruit preservation. Food Chem..

[3] Perumal A.B., Huang L., Nambiar R.B., He Y., Li X., Sellamuthu P.S. (2022). Application of essential oils in packaging films for the preservation of fruits and vegetables: A review. Food Chem..

[4] Khanal A., Ullah M.A., Joyce P., White N., Macnish A., Hoffman E., Irving D., Webb R., Joyce D. (2024). Impact of Fruit Maturity on Internal Disorders in Vapor Heat Treated Mango Cv. ‘B74’. Susabinibility.

[5] Zhong Y., Cui Y., Yu J., Bai J., Xu H., Li M. (2025). Electron-beam generated X-ray irradiation treatment alleviates fruit-body softening of harvested Hericium erinaceus by regulating metabolisms of membrane lipid and cell wall. Postharvest Biol. Technol..

[6] Balasubramanian P., Xiao H.W., Sutar P.P. (2025). Effect of cyclic vacuum-steam blanching on the quality characteristics and functional properties of Malabar spinach (Basella alba) dried by non-water infrared refractance window drying. Food Chem..

[7] Muthuvelu K.S., Ethiraj B., Pramnik S., Raj N.K., Venkataraman S., Rajendran D.S., Bharathi P., Palanisamy E., Narayanan A.S., Vaidyanathan V.K. (2023). Biopreservative technologies of food: An alternative to chemical preservation and recent developments. Food Sci. Biotechnol..

[8] Wu J., Zhang Y., Zhang F., Mi S., Yu W., Sang Y., Wang X. (2025). Preparation of chitosan/polyvinyl alcohol antibacterial indicator composite film loaded with AgNPs and purple sweet potato anthocyanins and its application in strawberry preservation. Food Chem..

[9] Bora N.S., Kalita P., Mary S.E., Roy S. (2025). Lipid-based food packaging film/coating in postharvest fruit preservation. Food Packag. Shelf Life.

[10] Ncube L.K., Ude A.U., Ogunmuyiwa E.N., Zulkifli R., Beas I.N. (2020). Environmental Impact of Food Packaging Materials: A Review of Contemporary Development from Conventional Plastics to Polylactic Acid Based Materials. Materials.

[11] Wang J., Yuan Y., Liu Y., Li X., Wu S. (2024). Application of chitosan in fruit preservation: A review. Food Chem. X.

[12] Iqbal Y., Ahmed I., Irfan M.F., Chatha S.A.S., Zubair M., Ullah A. (2023). Recent advances in chitosan-based materials; The synthesis, modifications and biomedical applications. Carbohyd. Polym..

[13] Li R., Liu Z., Guo J., Xu W., Ning X., Li X., He M., Liu Q. (2025). Active chitosan films crosslinked by vanillin-derived dialdehyde for effective fruit preservation. Food Hydrocolloid.

[14] Zhao J., Yang H., Li C., Xu Z., Shan P., Fu C., Tao Y., Hu J., Wang H., Du J. (2025). Eco-friendly chitosan-based composite film with anti-dissolution capacity as active packaging for fruit preservation. Food Hydrocolloid.

[15] Feng Y., Yang F., Yuan W., Hu C., Chu F., Wu Y., Xiong F. (2025). Lignin micro-nanospheres loaded with silver nanoparticles for excellent antibacterial activity. Int. J. Biol. Macromol..

[16] Tejamaya M., Römer I., Merrifield R.C., Lead J.R. (2012). Stability of Citrate, PVP, and PEG Coated Silver Nanoparticles in Ecotoxicology Media. Environ. Sci. Technol..

[17] Qin Z., Zheng Y., Wang Y., Du T., Li C., Wang X., Jiang H. (2021). Versatile roles of silver in Ag-based nanoalloys for antibacterial applications. Coord. Chem. Rev..

[18] Prabhu R., Devaraju A. (2018). Developing an Antimicrobial Packaging to Improve the Shelf Life of Meat Using Silver Zeolite Coating on BOPP Film. Mater. Today Proc..

[19] Yi J., Zhang S., Yuan X., Zhang Z., Shan Z., Wang H. (2024). Development of multifunctional zein-based films engineered with gallic acid and Ag NPs loaded γ-CD-MOFs for pork preservation. Food Packag. Shelf Life.

[20] Dai X., Li S., Li S., Ke K., Pang J., Wu C., Yan Z. (2022). High antibacterial activity of chitosan films with covalent organic frameworks immobilized silver nanoparticles. Int. J. Biol. Macromol..

[21] He Q., Zhang Y., Zhao W., Deng Y., Li H., Lin B. (2024). Super protective effect, ultra-high juice absorption and long-term antibacterial of Ag-2MI@Chitosan biodegradable sponge for fruit preservation and transportation. Int. J. Biol. Macromol..

[22] Zhang Z., Zhao Y., Hu Z., Si Z., Yang Z. (2023). 2-Pyridinecarboxaldehyde-Modified Chitosan–Silver Complexes: Optimized Preparation, Characterization, and Antibacterial Activity. Molecules.

[23] Gierszewska M., Jakubowska E., Richert A. (2023). The adenine-modified edible chitosan films containing choline chloride and citric acid mixture. Sci. Rep..

[24] (1997). Geometrical Product Specifications (GPS)—Surface Texture: Profile Method—Terms, Definitions and Surface Texture Parameters.

[25] Bodana V., Swer T.L., Kumar N., Singh A., Samtiya M., Sari T.P., Babar O.A. (2024). Development and characterization of pomegranate peel extract-functionalized jackfruit seed starch-based edible films and coatings for prolonging the shelf life of white grapes. Int. J. Biol. Macromol..

[26] Diridiri P.N., Bodur S.E., Bayraktar A., Günkara Ö.T., Bakırdere S. (2024). Determination of copper ion at trace levels in apple tea samples by simultaneous complexation and spray assisted microextraction method prior to detection by flame atomic absorption spectrophotometry. Food Chem..

[27] Sabzevari S., Farrokhzad H., Poorkhalil A. (2025). Synthesis and characterization of a hydrogel film based on chitosan/carboxymethyl cellulose for food packaging applications. Carbohyd. Polym. Technol. Appl..

[28] Jridi M., Hajji S., Ayed H.B., Lassoued I., Mbarek A., Kammoun M., Souissi N., Nasri M. (2014). Physical, structural, antioxidant and antimicrobial properties of gelatin–chitosan composite edible films. Int. J. Biol. Macromol..

[29] Chen H., Lan X., Guan X., Luo R., Zhang Q., Ren H., Xu Z., Tang J. (2024). Comparative study on the effects of chitosan, carrageenan, and sodium alginate on the film-forming properties of fish skin gelatin. LWT.

[30] Dong Y., Li Y., Ma Z., Rao Z., Zheng X., Tang K., Liu J. (2023). Effect of polyol plasticizers on properties and microstructure of soluble soybean polysaccharide edible films. Food Packag. Shelf Life.

[31] Ben Z.Y., Samsudin H., Yhaya M.F. (2022). Glycerol: Its properties, polymer synthesis, and applications in starch based films. Eur. Polym. J..

[32] Asfaw W.A., Tafa K.D., Satheesh N. (2023). Optimization of citron peel pectin and glycerol concentration in the production of edible film using response surface methodology. Heliyon.

[33] Leceta I., Guerrero P., de la Caba K. (2013). Functional properties of chitosan-based films. Carbohyd. Polym..

[34] Cerqueira M.A., Souza B.W.S., Teixeira J.A., Vicente A.A. (2012). Effect of glycerol and corn oil on physicochemical properties of polysaccharide films—A comparative study. Food Hydrocolloid.

[35] Nina M., Fathana H., Iqhrammullah M. (2022). Preparation and characterization of new magnetic chitosan-glycine-PEGDE (Fe3O4/Ch-G-P) beads for aqueous Cd(II) removal. J. Water Process Eng..

[36] Wang Z., Liu X., Ji J., Guo Y., Zhu Y., Zhang G., Tong B., Jiao Y., Liu K. (2024). Suppressed Droplet Splashing on Positively Skewed Surfaces for High-Efficiency Evaporation Cooling. Small.

[37] Gabriele F., Donnadio A., Casciola M., Germani R., Spreti N. (2021). Ionic and covalent crosslinking in chitosan-succinic acid membranes: Effect on physicochemical properties. Carbohyd. Polym..

[38] Mouzahim M.E., Eddarai E.M., Eladaoui S., Guenbour A., Bellaouchou A., Zarrouk A., Boussen R. (2023). Effect of Kaolin clay and Ficus carica mediated silver nanoparticles on chitosan food packaging film for fresh apple slice preservation. Food Chem..

[39] Chalitangkoon J., Wongkittisin M., Monvisade P. (2020). Silver loaded hydroxyethylacryl chitosan/sodium alginate hydrogel films for controlled drug release wound dressings. Int. J. Biol. Macromol..

[40] Zhang Q., Peng Y., Liu Z., Huang M., Bi X., Ahmad M., Hao G. (2026). Fabrication and physicochemical characterization of Litsea cubeba essential oil nanoemulsion/chitosan composite films for yak butter preservation. Food Chem..

[41] Ardean C., Davidescu C.M., Nemeş N.S., Negrea A., Ciopec M., Duteanu N., Negrea P., Duda-Seiman D., Musta V. (2021). Factors Influencing the Antibacterial Activity of Chitosan and Chitosan Modified by Functionalization. Int. J. Mol. Sci..

[42] Anush S.M., Vishalakshi B., Kalluraya B., Manju N. (2018). Synthesis of pyrazole-based Schiff bases of Chitosan: Evaluation of antimicrobial activity. Int. J. Biol. Macromol..

[43] Hamed A.A., Abdelhamid I.A., Saad G.R., Elkady N.A., Elsabee M.Z. (2020). Synthesis, characterization and antimicrobial activity of a novel chitosan Schiff bases based on heterocyclic moieties. Int. J. Biol. Macromol..

[44] Su J., Liu C., Sun A., Yan J., Sang F., Xin Y., Zhao Y., Wang S., Dang Q. (2025). Hemostatic and antimicrobial properties of chitosan-based wound healing dressings: A review. Int. J. Biol. Macromol..

[45] Yu R., de Saint-Cyr L.C., Soussan L., Barboiu M., Li S. (2021). Anti-bacterial dynamic hydrogels prepared from O-carboxymethyl chitosan by dual imine bond crosslinking for biomedical applications. Int. J. Biol. Macromol..

[46] Abdel-Baky Y.M., Ragab A., Abusaif M.S., Ammar Y.A., Omer A.M. (2025). Novel antibacterial, antioxidant, and anti-inflammatory aminated chitosan hybrid quinoline Schiff base as multi-target agent: Design, molecular docking, and toxicity assessment. Carbohyd. Polym..

[47] More P.R., Pandit S., Filippis A., Franci G., Mijakovic I., Galdiero M. (2023). Silver Nanoparticles: Bactericidal and Mechanistic Approach against Drug Resistant Pathogens. Microorganisms.

[48] Ali S., Bahadur A., Hassan A., Ahmad S., Shah W., Iqbal S. (2025). Optimized silver nanostructures for enhanced antibacterial potential: Recent trends and challenges in the development of metallo-antimicrobials. Chem. Eng. J..

[49] Sathiyaseelan A., Zhang X., Kumaran S., Wang M.-H. (2025). Chitosan fabricated silver nitroprusside nanocomposites prepared for enhanced antibacterial and cytocompatibility applications through controlled release of metal ions and nitric oxide. Int. J. Biol. Macromol..

[50] Gol N.B., Patel P.R., Rao T.V.R. (2013). Improvement of quality and shelf-life of strawberries with edible coatings enriched with chitosan. Postharvest Biol. Tec..

[51] Qian L., Jia R., Zhao Q., Sun N., Yang J., Wen J., Li H., Yang J., Mo L., Gao W. (2025). Tough, antibacterial, and antioxidant chitosan-based composite films enhanced with proanthocyanidin and carvacrol essential oil for fruit preservation. Food Res. Int..

[52] Liu X., Sun X., Du H., Li Y., Wen Y., Zhu Z. (2024). A transparent p-coumaric acid-grafted-chitosan coating with antimicrobial, antioxidant and antifogging properties for fruit packaging applications. Carbohyd. Polym..

[53] Xu W., Liu K., Li T., Zhang W., Dong Y., Lv J., Wang W., Sun J., Li M., Wang M. (2019). An in situ hydrogel based on carboxymethyl chitosan and sodium alginate dialdehyde for corneal wound healing after alkali burn. J. Biomed. Mater. Res. A.

[54] Lu N., Chen Z., Zhang W., Yang G., Liu Q., Böttger R., Zhou S., Liu Y. (2021). Effect of silver ion implantation on antibacterial ability of polyethylene food packing films. Food Packag. Shelf Life.

